# Reshaping the full body illusion through visuo-electro-tactile sensations

**DOI:** 10.1371/journal.pone.0280628

**Published:** 2023-02-01

**Authors:** Greta Preatoni, Francesca Dell’Eva, Giacomo Valle, Alessandra Pedrocchi, Stanisa Raspopovic

**Affiliations:** 1 Department of Health Sciences and Technology, Laboratory for Neuroengineering, Institute for Robotics and Intelligent Systems, ETH Zürich, Zürich, Switzerland; 2 NearLab, Department of Electronics Information and Bioengineering and We-Cobot Interdept, Lab, Politecnico di Milano, Milano, Italy; Anglia Ruskin University, UNITED KINGDOM

## Abstract

The physical boundaries of our body do not define what we perceive as *self*. This malleable representation arises from the neural integration of sensory information coming from the environment. Manipulating the visual and haptic cues produces changes in body perception, inducing the Full Body Illusion (FBI), a vastly used approach to exploring humans’ perception. After pioneering FBI demonstrations, issues arose regarding its setup, using experimenter-based touch and pre-recorded videos. Moreover, its outcome measures are based mainly on subjective reports, leading to biased results, or on heterogeneous objective ones giving poor consensus on their validity. To address these limitations, we developed and tested a multisensory platform allowing highly controlled experimental conditions, thanks to the leveraged use of innovative technologies: Virtual Reality (VR) and Transcutaneous Electrical Nerve Stimulation (TENS). This enabled a high spatial and temporal precision of the visual and haptic cues, efficiently eliciting FBI. While it matched the classic approach in subjective measures, our setup resulted also in significant results for all objective measurements. Importantly, FBI was elicited when all 4 limbs were multimodally stimulated but also in a single limb condition. Our results behoove the adoption of a comprehensive set of measures, introducing a new neuroscientific platform to investigate body representations.

## Introduction

Every day we experience an incredible number of stimuli from the world. Our senses can integrate this information in a process known as multisensory integration [1, 2], which allows us to come up with a unitary perception of reality [3] and, most importantly, a representation of what we consider our “self” [2, 4]. Even if these mechanisms are automatic and often taken for granted, several neurological diseases (e.g., stroke [5], somatoparaphrenia [6]) lead patients to perceive this coherent self-representation as altered and even to deny parts of their body.

Interestingly, researchers have discovered that it is possible to experimentally manipulate body ownership in healthy subjects. The first evidence goes back to 1998 when Botvinick and Cohen proposed the Rubber Hand Illusion (RHI) paradigm [7]: if an experimenter strokes the real hand of a subject (occluded from his sight) while performing the same synchronous movement on a rubber hand in his sight, he/she will be tricked into believing that the rubber hand is his/her own. The reason why this happens is that the sense of body ownership arises thanks to the coherence of simultaneous sensory input combined with a pre-existing cognitive representation of the body [8, 9]. Indeed, if the RHI is performed with visuo-tactile stimuli administered asynchronously, the illusion fails to take place.

The discovery that our body representation is malleable and that the spatial limits of this bodily self-consciousness can go beyond our physical body has led scientists to explore to what extent this applies. Indeed, later studies found that manipulating the spatio-temporal congruency of sensory inputs can induce the same illusion also for the lower limb (Rubber Foot Illusion) [10] and on a full-body level (Full Body Illusion—FBI) [11]. During the FBI, the subject sees a mannequin in front of him receiving a stimulation on the back, while he is being stroked in the same location on his real body [12]. Numerous researchers have replicated this illusion with slight changes to understand what are the necessary and sufficient conditions for the illusion to arise [12, 13]. This has led to the general consensus on the importance of the time synchronicity between the multisensory stimulation and of their spatial congruency [12, 14], but the best conditions under which the illusion happens are still not completely clear.

Indeed, many of the studies only use subjective reports that have the drawback of not confidently ruling out the suggestibility of subjects [15], compromising the interpretation of the results. Indeed, there is a debate on the fact that the *a priori* expectations of the subjects might be influenced by the specific questions of the questionnaires [15]. To objectively measure the strength of the illusion, one of the first proposed methods was the Proprioceptive Drift (PD) [11, 16, 17] defined as *a mislocalization in the perceived location of the real hand/body towards the fake one*. Even though this method has frequently been used, some studies have suggested that it does not correlate with the illusion [18]. For the FBI paradigms, the classical method implied that at the end of the experiment the subject (blindfolded) was moved a few meters away from his/her position and then asked to return to the previous location (locomotion task (LT)). This method has been criticized as the LT could update the somatosensory and vestibular signals of the subject, making it hard to maintain the illusory self-location [16]. To overcome this, recently a new method based on a mental imagery task (MIT) has been proposed where the subject localizes himself in relation to a virtual ball approaching him [16], by pressing a button when he thinks that the ball reached his feet.

Another objective measure proposed to correlate with the illusion is the peripersonal space (PPS) [19, 20]. PPS is defined as *the space immediately surrounding our bodies* [21], *where human-environment interactions take place through multisensory integration* [1]. Subjects react faster to two stimuli presented at the same time (e.g. vision and touch) if they surpass the borders of the PPS (i.e., closer to the subject) [22]. Notably, it has been proven that after synchronous multisensory stimulation to induce the FBI, the boundaries of the PPS extend in the front-space, shifting towards the fake body [19]. Unfortunately, the vast heterogeneity in the approaches to measure the illusion makes it difficult to quantitatively compare the results between studies and to shed light on the validity of the metrics.

**Fig 1:**
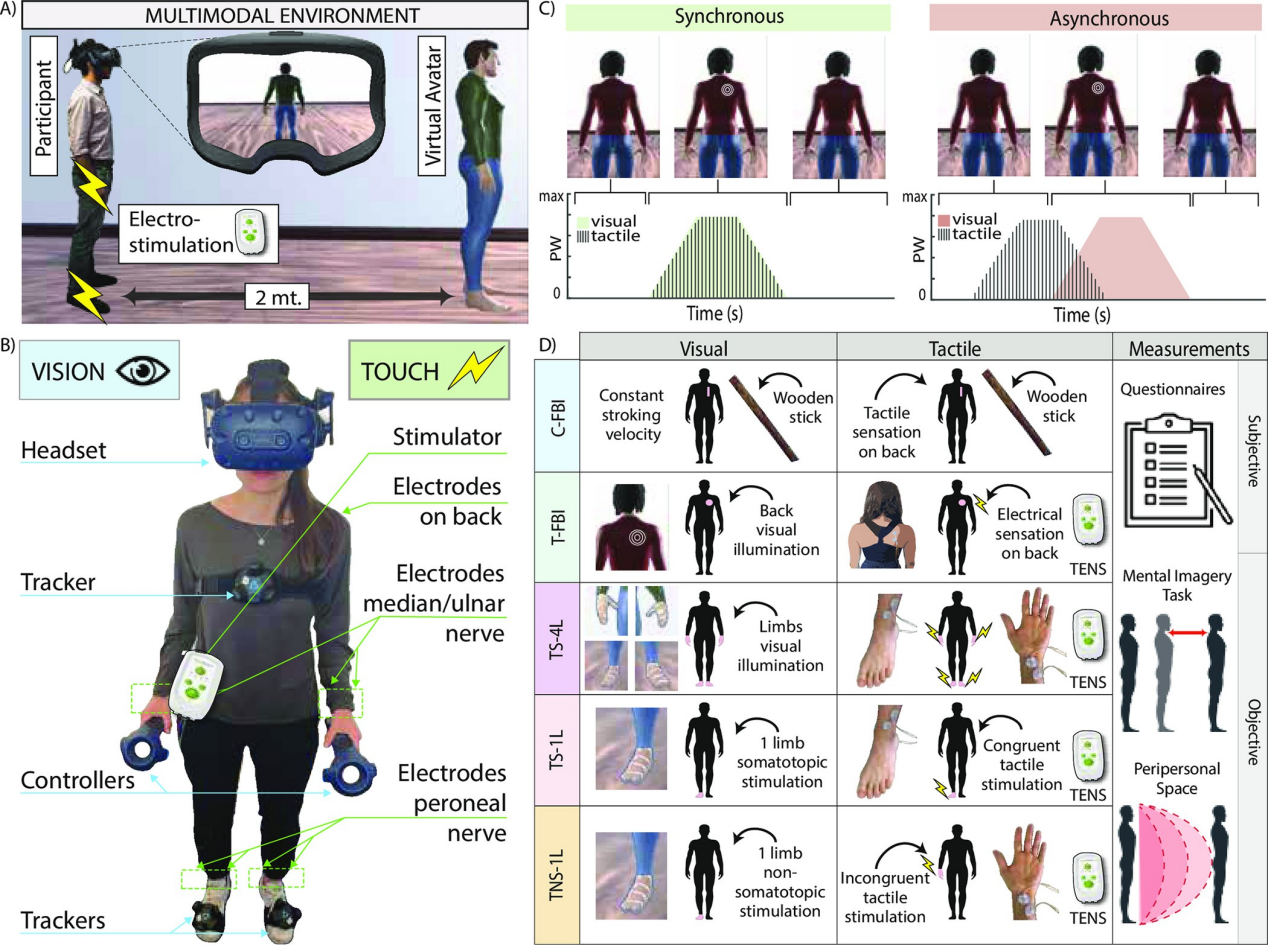
Experimental setup and procedure. A) Virtual environment. The subject was placed 2 meters apart from the virtual mannequin and saw him from the back. B) Set-up. The subject wore a head-mounted display, held controllers in his/her hands, had trackers on the chest and feet, and had electrodes placed on the feet, hands, and back. C) The electrical stimulation could be either synchronous (left) to the visual stimulus or asynchronous (right). D) Experimental conditions and outcomes. Every row represents a different condition (first column). The second and third columns show the visual and tactile stimulation respectively, for each condition. The fourth column shows the outcome measurements. C-FBI = classical FBI; T-FBI = TENS FBI; TS-4L = TENS on 4 limbs somatotopic; TS-1L = TENS on 1 limb somatotopic; TNS-1L = TENS on 1 limb non-somatotopic.

In this study, we decided to develop a multisensory technology able to provide visual feedback through Virtual Reality (VR) and somatosensory feedback through Transcutaneous Electrical Nerve Stimulation (TENS) (Fig 1A and 1B). This platform has the scope of inducing the FBI with a more accurate and controlled technology allowing a high precision in the generation and the assessment of the illusion. Importantly, we applied a multifaceted evaluation approach with complete, objective, and subjective measurements. In particular, we adopted: 1) validated subjective self-experience questionnaires 2) MIT measured with the recently proposed mental imagery task (MIT) and 3) PPS.

VR is increasingly considered a useful tool in cognitive neuroscience and physiology and is used to replicate the FBI [23]. However, its integration with various technologies, necessary for virtual embodiment, can be daunting [23]. In particular, somatosensory feedback should be carefully chosen based on several important characteristics [24]. First of all, given that our skin has numerous and specialized touch receptor cells, the haptic technology should be decided based on the target application and take into account the safety of the participant [23]. Moreover, ergonomics have to be considered: the device should be light and allow the subject to freely move during the experiment [23]. Up until today, the tactile stimulus has mainly been administered in an experimenter-based manner [11], through visuo-tactile actuators [25] or robotic systems [26]. However, these approaches have several limitations. Firstly, experimenter-based delivery of tactile stimuli has the limitation of not having a high and constant control on the precision of time and location synchronicity and of not being able to adapt to the subject’s movements. Secondly, vibrotactile actuators have the capacity of eliciting a sensation in very small pre-fixed locations (i.e., many vibrators need to be used) which are limited to vibration-like sensations and have drawbacks in terms of miniaturization and power consumption. Finally, robotic systems using mechano-tactile feedback allow a precise time and location synchronicity but are difficult to be transformed into small and low-power devices to be used in clinical settings, and clearly cannot adapt to the subject’s movements.

Our solution integrating VR with TENS can overcome these limitations: a head-mounted display immerses the subject in a realistic environment, where visual stimuli are synchronized in a millisecond precision with the somatosensory feedback delivered through couples of electrodes, able to stimulate multiple body parts concomitantly. Alongside the ability to be extremely temporally precise with the visual stimulus, the advantages of electrical stimulation are several. The electrical stimulator can easily be placed on the subject’s body and further away from the electrodes, allowing a great range of movement and comfort. Furthermore, it has low activation delays because it does not need to overcome the inertia of moving components. Finally, it can elicit somatotopic sensations, impossible with other methodologies: with a proper electrode placement on the limbs, TENS can directly activate neural structures, leading to referred sensations in areas distal to where the electrodes are placed (somatotopic sensations) [27]. This peculiarity becomes significant especially when considering a rehabilitation application in patients who suffer from sensory loss and the possibility of eliciting sensations in body areas that they can no longer feel.

To validate the proposed integrated multisensory approach, we first performed a classical FBI paradigm, where the subject was immersed in the VR environment watching a mannequin stroked on its back and feeling a stroke on his/her body in the same location, performed by the experimenter. The condition was tested both in synchronous and asynchronous cases. Then, other 2 conditions were performed, keeping the same stimuli location (i.e., back of the trunk) but delivering the somatosensory feedback through TENS and using a homologous stimulation (i.e., illumination of the targeted area) as visual feedback. We hypothesized that similar to the classical FBI, also the VR-TENS approach on the trunk would have resulted in a significantly higher embodiment for the synchronous case.

In a second phase, we tested whether it was also possible to induce the FBI with a somatotopic electrical stimulation. In this case, electrodes were placed on the median and peroneal nerves to elicit a sensation spreading distally on the upper and lower limbs. Previous studies have shown that it is possible to induce the RHI in upper-limb amputees eliciting somatotopic sensations. However, sensations were elicited with an experimenter-based brush, which has the above-mentioned limitations, and it has never been shown on an FBI level. Therefore, scientific validation of a somatotopic FBI with healthy volunteers is the first step to open up the possibility of recreating such an illusion also on populations with sensory deficits.

Furthermore, given the knowledge that the subjective ownership of a mannequin’s body arises irrespective of which singular body part is being stimulated [28–30], we wanted to assess if this was true also for our approach. Therefore, we investigated this “spread of embodiment” with a fourth condition where the TENS and visual stimulation were applied to only one limb (i.e., foot).

Finally, to check whether the spatial congruency of the visual and tactile stimuli was a necessary condition, we performed a last control condition where we applied the TENS stimulation to one limb and the visual to another one.

## Materials and methods

### Subjects

22 healthy subjects took part in the experiment (11 males and 11 females, mean age of 27). The study was approved by the Ethic Commission of ETH Zürich (EK 2020-N-113) and all participants signed an informed consent form. The pictures and videos used in the study come from participants who allowed in the written consent form to publish the figures and videos generated in this study to be used in an online open-access publication. All experiments were performed in accordance with the Declaration of Helsinki.

### Experimental procedure

Participants were immersed in a Virtual Reality (VR) environment, finding themselves in a room with a character (avatar) in front of them, seen from its back (3^rd^ person perspective) (Fig 1A). The distance between the subject and the avatar was 2 meters. The congruency between real and virtual distance was checked and set knowing that 1 VR unit corresponds to 1 meter in the real world. The avatar could represent either a man or a woman and its height was 1.80 meters by default but scalable.

The virtual scenario was implemented with Unity 3D (Unity Technologies, United States). Its rendering was realized using the VR equipment by HTC Vive Pro Eye (Valve Corporation, United States). It comprises a head-mounted display (HMD) which was head tracked, 2 controllers held by the participant for both hands tracking and to interact with the program, 2 trackers on both feet, 1 on the chest, and 2 base stations to coordinate the communication and tracking of the other devices (Fig 1B, S1 Video).

The experiment was run as a series of multiple conditions in a randomized order separated by *10 minutes* of pause.

Each condition had a duration of approximately 20 minutes during which 60 seconds of stimulation to induce the illusion were alternated with the registration of an outcome measure done in the virtual environment (S1 Fig in S1 File). This pattern was used to acquire the measurements while the illusion was still vivid and not fade out. It was repeated until all metrics were acquired (i.e., three times) (see S1 File).

The total number of subjects (22) was divided into equally sized groups: one group performed two conditions (classical FBI (C-FBI) and TENS somatotopic on 4 limbs (TS-4L)) and one group performed three conditions (TENS on back (T-FBI), TENS 1 limb somatotopic (TS-1L), TENS 1 limb non-somatotopic (TNS-1L)). The decision of dividing subjects in 2 groups rather than having everyone doing all conditions was done to avoid fatigue and to adopt a stronger between-subject design.

Each condition consisted in a synchronous visuo-tactile stimulation where the two stimuli are provided at the same time and its relative control, hence an asynchronous one with a time mismatch between the two stimuli (see S1 File) (Fig 1C) (S1 Video).

The visuo-tactile stroking differed between the conditions (Fig 1D). In the reproduction of the C-FBI experiment, participants were stroked on their backs with a stick while looking at a similar object moving over the avatar’s back. The other conditions employed the use of TENS instead of the stick to reproduce the tactile stimulation with a matching visual stimulus consisting of white moving lines (T-FBI, Fig 1D). The attempt was to physically render the electrical stimulation with something that could resemble its somatosensory sensation. The first condition tested with this novel approach (T-FBI) matched the location of stimulation of the C-FBI: participants received an electrical stimulation on the back while seeing the visual illumination (resembling the perceived electrical sensation) on the avatar’s back. Then, a similar stimulation was used on all the limbs (TS-4L): electrodes were placed on the median and peroneal nerves to elicit somatotopic sensations distally on the hands and feet. Finally, two conditions were tested by stimulating just one limb: one condition had spatial congruency between the visuo-tactile stimulation provided on the right foot (TS-1L), and the other, instead, had no spatial congruency (TNS-1L), with the visual stimulation provided to the foot and the tactile one to the hand.

### Sensation calibration procedure

The electrical stimulator (RehaMove, HASOMED GmbH, Germany) was controlled with ad-hoc software which allowed the setting of current amplitude, frequency, and pulsewidth.

Couples of superficial electrodes (circle Pads, Ø = 25mm, TensCare, England) were placed on the subjects. Their placement was either on the back trunk (T-FBI), on the median/ulnar nerve (TS-4L, TS-1L, TNS-1L), or the peroneal nerve (TS-4L, TS-1L) (Fig 1B) (see S1 File).

To find the proper stimulation values for each participant a calibration procedure was performed before the experiment. First, trains of biphasic balanced stimuli with a ramp of increasing amplitude, and fixed pulsewidth (300 us), lasting 2 seconds with 1 second of pause were delivered. It was used to find the amplitude at which the artificial sensation was perceived as comfortable and somatotopic for the interested limbs. Once found the proper amplitude, a ramp on pulsewidth was performed to find this parameter. Here, subjects had to inform when they felt both a light sensation (intensity = 2/10) and a strong one but still not painful (intensity = 8/10). These values were chosen to be able to modulate the intensity of the perceived location which would result in a higher area where the subject could perceive the sensation, resembling the visual illumination of the corresponding body part. Each ramp was repeated three times and the means of these values were taken as minimum and maximum pulsewidth and used in the experiment. Frequency was kept always constant at 50 Hz [31–33].

After each ramp, participants completed a form regarding the felt sensation. Using an iPad, they were asked to color over an image (S2 Fig in S1 File) of the calibrated body part where they felt the sensation and to indicate its perceived intensity and type. This procedure was repeated for each body location stimulated.

### Outcome FBI measures

#### Questionnaires

After each condition, subjects answered questionnaires regarding the FBI. These standard questionnaires include questions from the embodiment questionnaire [34, 35] (6 questions with their controls and 2 questions on vividness and prevalence of the illusion, S1 Table in S1 File) and from the Phenomenology of Consciousness Inventory Questionnaire (PCI, S3 Table in S1 File) [36] (3 questions related to the subscale of body image, see S1 File). All these items were presented each time in random order to the participant. This set of questions was implemented and answered in the VR environment using the controller in order not to distract participants with stimuli from the external world. Answers were given on a 7-points scale, from -3 (completely disagree) to 3 (completely agree), then shifted in the Results section from 0 to 6.

#### Peri-personal space (PPS)

To compute the peripersonal space (PPS), touch and visual stimuli were used [1]: the former consisted of electrical pulses (lasting *100 ms*) delivered to the subject, the latter of a tennis ball looming towards him/her in the VR environment (S3 Fig in S1 File). Participants were instructed to press a button as soon as they felt the pulse while looking at the approaching ball (S1 Video). The electrical input could be given when the ball was at six different distances from the subjects and the Reaction Times (RTs) were collected (see S1 File).

Two different types of PPS were measured, namely peri-trunk and peri-foot, differing in both ball and electrical pulses location. In case the ongoing condition included either a back or a 4 limbs stimulation, the former PPS was applied, with the ball looming towards the subject’s face and the pulses delivered to his hand. Otherwise, when the condition comprised a foot stimulation, the latter PPS was used, with the ball looming towards the subject’s foot and the pulses delivered to this same limb (see S1 File).

PPS boundary was computed as the position where subjects became significantly faster than baseline, meaning that multisensory integration was occurring. The baseline measure for each condition was retrieved by doing several trials only with the electrical pulses, without any visual cue.

In addition to these measurements, also catch trials were carried out: in this case the ball was looming towards the participant, but he/she was not receiving a tactile stimulation and so was not expected to press the button. Their aim was to test whether the subject was continuously focused on the required task.

The total number of runs (for each condition) was: 60 experimental trials (10 for each distance), 20 baseline trials (only measured for D1 and D6), and 10 catch trials.

#### Mental imagery task

To compute the “perceived self-localization” a recently proposed method [16] was used: the avatar was removed from the scene and a red ball appeared, rolling on the floor towards the subject. After 3 seconds, the HMD screen became black and the participant was instructed to press a button on the VR controller when he/she imagined the ball passing through his/her feet (S1 Video). The position of the ball when the button was pressed was taken as a proxy of the perceived self-location (see S1 File).

### Statistical analysis

Statistical analysis was performed using Matlab2019 (MathWorks, United States). The aim was to assess whether different conditions had a significant effect on data collected from various metrics. Both between and within-subjects analyses were performed according to the data to be analyzed. Effect sizes and statistical power were performed using G*Power. All alpha levels were set at 0.05.

For questionnaires and MIT results, the normality of data was checked using the one-sample Kolmogorov-Smirnov test. Then, for double-fold comparisons, a t-test was used in the case of normally distributed data while a Wilcoxon Signed Rank test was carried out for non-normal ones. In both cases, conditions were considered significantly different when *p*<0.05.

For PPS results, a 2 steps analysis was designed. Firstly, a 1 (synchronicity) x 6 (Distances) within-subjects ANOVA was conducted on different RTs, to test the main effect of the 2 variables. Secondly, paired t-tests were carried out for every condition to compare the RT of each sound distance with the condition-specific baseline of the participant. Indeed, for each subject, a single condition-specific baseline was considered.

Baseline acquisitions (with electrical pulse but without the ball) were done in each condition only at 2 distances (D1 and D6); then, the slower between the two (most conservative approach) was taken as the baseline of that specific condition for that subject [19].

## Results

### Electrically-induced sensations as somatosensory stimuli

**Fig 2:**
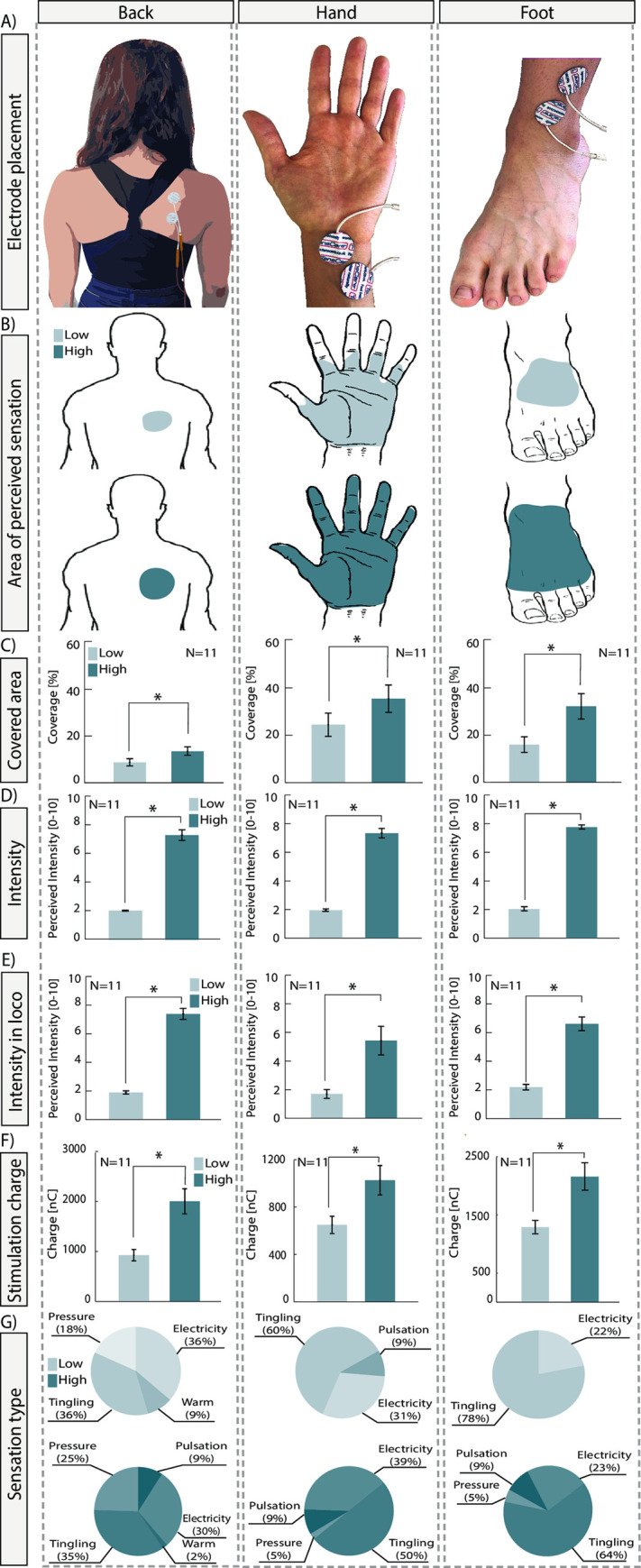
Results of the calibration procedure. For each stimulated location (back trunk, wrists, and ankles in column), the following information is reported for the low (light blue) and high (dark blue) intensity: A) electrodes placement; B) Area of perceived location; C) Covered area in percentage; D) Intensity of the perceived sensation; E) Intensity in loco; F) Stimulation charge used; G) Sensation type reported. Significant results are reported when p<0.05. The error bars represent the SEM.

Results derived from the questionnaire held during the calibration procedure are summarized in Fig 2.

Electrodes were placed on the back trunk, wrist, and ankle (Fig 2A). As expected, the area of perceived stimulation increased with higher stimulation intensity (Fig 2B). Indeed, the percentage of the area covered by the evoked sensation (Fig 2C) had a significant difference (Wilcoxon Signrank, p-Value <0.001) between the low and the high case in all locations (Back_LOW_ = 8.67 ± 1.54%, Back_HIGH_ = 13.34 ± 1.8%, Hand_LOW_ = 24.41 ± 4.92%, Hand_HIGH_ = 35.44 ± 5.79%, Foot_LOW_ = 15.91 ± 3.29%, Foot_HIGH_ = 32.1 ± 5.37%). The perceived intensity (Fig 2D) had a significant difference between the low and the high stimulation for all locations (Wilcoxon sign rank, p-Value<0.001, Back_LOW_ = 2.03 ± 0.03, Back_HIGH_ = 7.29 ± 0.37, Hand_LOW_ = 1.95 ± 0.09, Hand_HIGH_ = 7.32 ± 0.33, Foot_LOW_ = 2.03 ± 0.05, Foot_HIGH_ = 7.76 ± 0.15). The intensity experienced directly under the electrodes (Fig 2E) showed, as well, a significant difference between the low and the high stimulation both for hands (Wilcoxon sign rank, p-Value = 0.004, Hand_LOW_ = 1.67 ± 0.3, Hand_HIGH_ = 5.33 ± 0.99) and for feet and back (Wilcoxon sign rank, p-Value <0.001, Back_LOW_ = 1.88 ± 0.11, Back_HIGH_ = 7.33 ± 0.38, Foot_LOW_ = 2.19 ± 0.19, Foot_HIGH_ = 6.61 ± 0.47). This was reflected in the actual charge (Fig 2F) delivered for the minimum and maximum levels of stimulation (Wilcoxon sign rank, p-Value<0.001, Back_LOW_ = 926.4 ± 114.36 nC, Back_HIGH_ = 2003.6 ± 250.97 nC, Hand_LOW_ = 649.1 ± 73.52 nC, Hand_HIGH_ = 1026.4 ± 124.16 nC, Foot_LOW_ = 1290.9 ± 114.32 nC, Foot_HIGH_ = 2157.3 ± 230.06 nC). The reported sensations for participants were never painful and were mostly tingling and electricity (Fig 2G).

### FBI is induced by electrical neurostimulation

**Fig 3:**
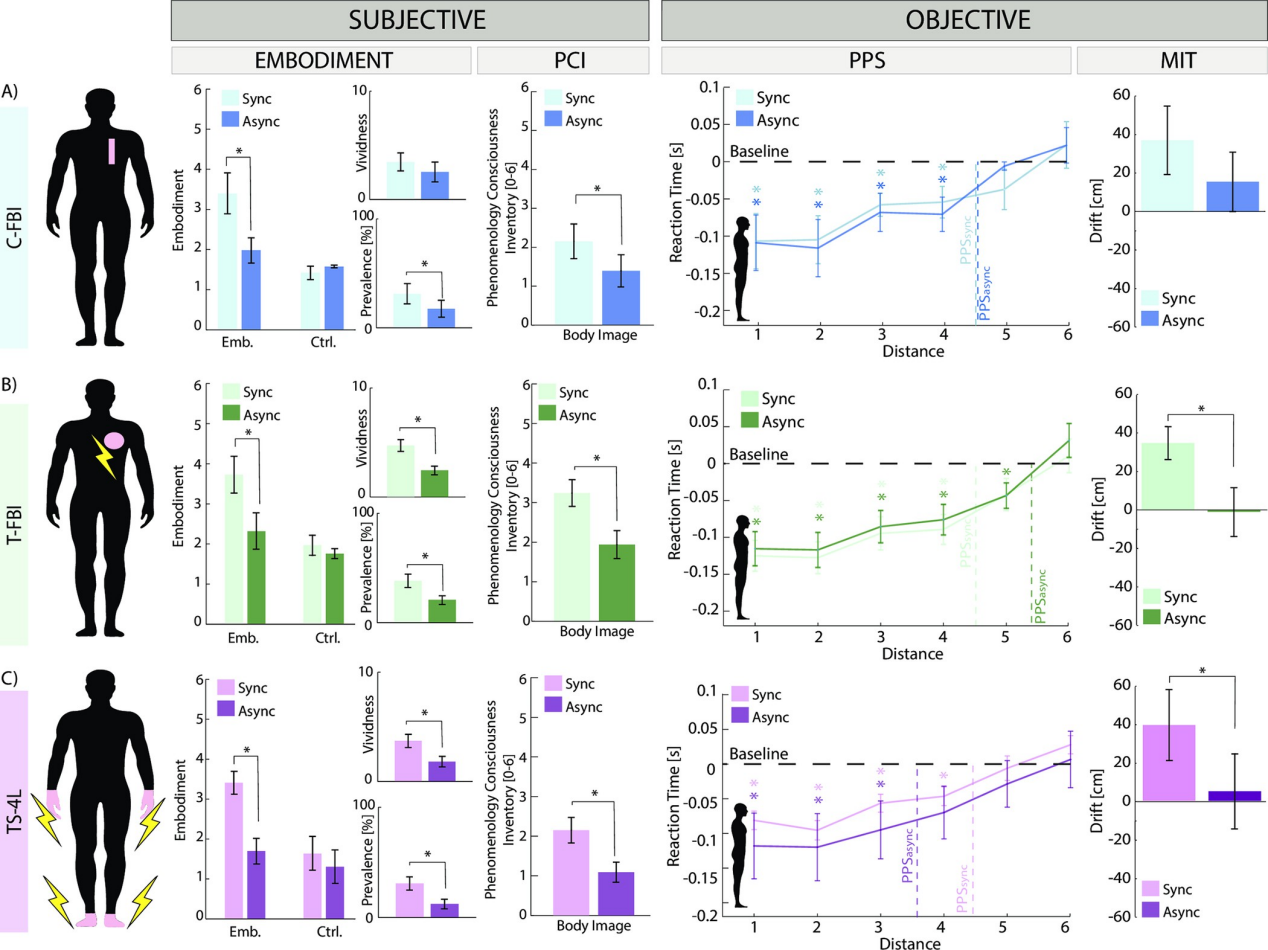
Results of experimental conditions (Classical-FBI (C-FBI), TENS-FBI (T-FBI), TENS-Somatotopic 4 Limbs (TS-4L)). Each panel is relative to a different condition: A) C-FBI, B) T-FBI, C) TS4L. For each condition, the following information is reported for the synchronous (light color) and asynchronous (dark color) alternative: location of the visuo-tactile stimulation, Questionnaires results (embodiment, vividness, prevalence, and body image), PPS results, and MIT results. *Significant results are reported when p<0.05. The error bars represent the SEM. C = classical FBI; T = TENS FBI; T4S = TENS on 4 limbs somatotopic.

In the classical FBI condition (C-FBI) (Fig 3A) the synchronous condition was rated as significantly higher compared to the asynchronous one for the embodiment questionnaire (Emb_SYNC_ = 3.39 ± 0.5, Emb_ASYNC_ = 1.98 ± 0.32, Wilcoxon sign rank p = 0.005, effect size dz (ES) = 3.2, statistical power = 1), for the illusion prevalence (Prev_SYNC_ = 31.18 ± 9.19, Prev_ASYNC_ = 17.36 ± 7.69, Wilcoxon sign rank p = 0.006, statistical power = 0.9) and for the body image (BI_SYNC_ = 2.15 ± 0.45, BI_ASYNC_ = 1.39 ± 0.41, Wilcoxon sign rank p = 0.03, statistical power = 0.9).

For the PPS, baseline corrected reaction times (RTs) were given to a 1 (condition) x 6 (distance 1 to 6) Kruskal-Wallis, which was significant for both the synchronous (H (5) = 24.9, p_SYNC_<0.001) and asynchronous (H (5) = 21.36, p_ASYNC_<0.001) case. Then, to identify the boundary of the PPS, post hoc multiple comparisons were run between each distance and the fastest RT of the unimodal tactile baseline. For both conditions, RTs to tactile stimuli for distances D1 to D4 were all significantly (all p<0.05) faster than the baseline, indicating that the boundary of the PPS was located between D4 and D5 for both conditions. All catch trials were never more than 2, indicating an appropriate accuracy of the subjects in detecting the tactile stimulus (S5 Fig in S1 File).

The Mental Imagery Task (MIT) did not show a significant difference between the conditions (MIT_SYNC_ = 0.37 ± 0.18 m, MIT_ASYNC_ = 0.15 ± 0.14 m, t-test p = 0.15).

Fig 3B shows the results for the paradigm repeated on the same location (back trunk) but with the tactile stimulus replaced with TENS. A significantly higher rate was found for all questionnaire items, hence the embodiment (Emb_SYNC_ = 3.73 ± 0.46, Emb_ASYNC_ = 2.32 ± 0.45, Wilcoxon sign rank p = 0.002, ES = 3.1, statistical power = 1), the vividness (Viv_SYNC_ = 4.73 ± 0.54, Viv_ASYNC_ = 2.45 ± 0.37, Wilcoxon sign rank p = 0.004, statistical power = 1), the prevalence (Prev_SYNC_ = 38.27 ± 6.16, Prev_ASYNC_ = 20.82 ± 3.82, Wilcoxon sign rank p = 0.012, statistical power = 1), and the body image (BI_SYNC_ = 3.24 ± 0.34, BI_ASYNC_ = 1.94 ± 0.35, Wilcoxon sign rank p = 0.019, statistical power = 1). For the PPS, the 1 (condition) x 6 (distance 1 to 6) Kruskal-Wallis was significant for both the synchronous (H (5) = 21.61, p_SYNC_<0.001) and asynchronous (H (5) = 34.98, p_ASYNC_<0.001) case. The posthoc multiple comparisons placed the boundary of the PPS for the asynchronous case between D5 and D6 (from D5 all p_async_< 0.03, ES = 3.6, statistical power = 1) and between D4 and D5 for the synchronous one (from D4 all p_sync_<0.0013, ES = 2.2, statistical power = 0.9). All catch trials were never more than 2, indicating an appropriate accuracy of the subjects in detecting the tactile stimulus (S5 Fig in S1 File). The MIT showed a significant trend of the synchronous condition in inducing a stronger drift (MIT_SYNC_ = 0.35 ± 0.08 m, MIT_ASYNC_ = -0.013 ± 0.12 m, Wilcoxon sign rank p< 0.001, ES = 3.4, statistical power = 1).

The 4 limbs visuo-electrical stimulation condition (TNS-4L) gave significant results in all subjective and objective measurements, in favor of the synchronous condition (Fig 3C). Indeed, all questionnaires showed a higher sense of embodiment for the synchronous case: embodiment (Emb_SYNC_ = 3.41 ± 0.29, Emb_ASYNC_ = 1.69 ± 0.32, Wilcoxon sign rank p = 0.002, ES = 5.6, statistical power = 1), vividness (Viv_SYNC_ = 3.73 ± 0.59, Viv_ASYNC_ = 1.82 ± 0.48, Wilcoxon sign rank p = 0.008, statistical power = 1), prevalence (Prev_SYNC_ = 30.90 ± 6.04, Prev_ASYNC_ = 12.36 ± 4.49, Wilcoxon sign rank p = 0.016, statistical power = 1), and body image (BI_SYNC_ = 2.15 ± 0.32, BI_ASYNC_ = 1.09 ± 0.25, Wilcoxon sign rank p = 0.002, statistical power = 1). The 1x6 Kruskal-Wallis on the PPS results reported a significant result for both the synchronous (H (5) = 26.93, p_SYNC_<0.001) and asynchronous (H (5) = 24.05, p_ASYNC_<0.001) case. The posthoc multiple comparisons placed the boundary of the PPS for the asynchronous case between D3 and D4 (from D3 all p_async_< 0.04, ES = 1.1, statistical power = 0.9) and between D4 and D5 for the synchronous one (from D4 all p_sync_< 0.006, ES = 0.8, statistical power = 0.7). All catch trials were never more than 3, indicating an appropriate accuracy of the subjects in detecting the tactile stimulus (S5 Fig in S1 File). Furthermore, also the MIT showed a significantly higher trend of the synchronous condition in inducing a stronger drift (MIT_SYNC_ = 0.39 ± 0.18 m, MIT_ASYNC_ = 0.05 ± 0.19 m, Wilcoxon sign rank p = 0.03, ES = 1.8, statistical power = 0.9).

**Fig 4:**
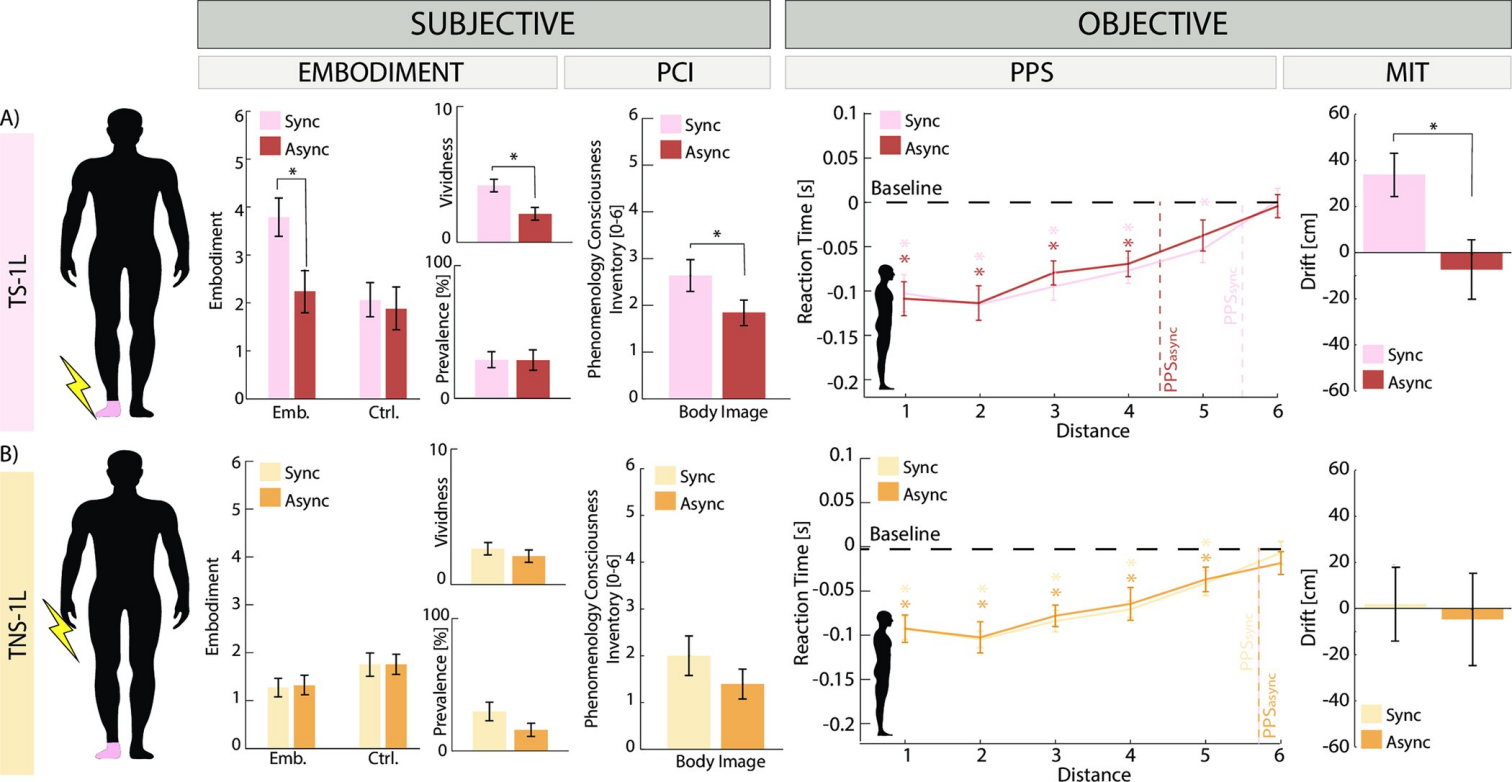
Results of experimental conditions (TS-1L, TNS-1L). Each panel is relative to a different condition: A) TS-1L, B) TNS-1L. For each condition, the following information is reported for the synchronous (light color) and asynchronous (dark color) alternative: location of the visuo-tactile stimulation, Questionnaires results (embodiment, vividness, prevalence, and body image), PPS results, and MIT results. Significant results are reported when p<0.05. The error bars represent the SEM. TS-1L = TENS on 1 limb somatotopic: TNS-1L = TENS on 1 limb non-somatotopic.

The last two conditions consisted of the stimulation of only one limb, either somatotopic (TS-1L) (Fig 4A) or not (TNS-1L) (Fig 4B).

In the somatotopic condition (TS-1L), significantly higher rates were reported in the synchronous case for embodiment (Emb_SYNC_ = 3.79 ± 0.39, Emb_ASYNC_ = 2.24 ± 0.44, Wilcoxon sign rank p = 0.03, ES = 3.7, statistical power = 1), vividness (Viv_SYNC_ = 4.18 ± 0.46, Viv_ASYNC_ = 2.09 ± 0.45, Wilcoxon sign rank p = 0.02, statistical power = 1) and body image (BI_SYNC_ = 2.64 ± 0.34, BI_ASYNC_ = 1.85 ± 0.27, Wilcoxon sign rank p = 0.01, statistical power = 1) but not for prevalence (Prev_SYNC_ = 29.00 ± 5.79, Prev_ASYNC_ = 28.73 ± 7.50, Wilcoxon sign rank p = 0.26). The 1x6 Kruskal-Wallis on the PPS results reported a significant result for both the synchronous (H (5) = 30.07, p_SYNC_<0.001) and asynchronous (H (5) = 23.27, p_ASYNC_<0.001) case. The posthoc multiple comparisons placed the boundary of the PPS for the asynchronous case between D4 and D5 (from D4 all p_async_<0.001, ES = 1.9, statistical power = 1) and between D5 and D6 for the synchronous one (from D5 all p_sync_<0.006, ES = 4.7, statistical power = 1). All catch trials were never more than 2, indicating an appropriate accuracy of the subjects in detecting the tactile stimulus (S5 Fig in S1 File). Lastly, MIT was significantly higher in the synchronous alternative (MIT_SYNC_ = 0.34 ± 0.09 m, MIT_ASYNC_ = -0.07 ± 0.13 m, Wilcoxon sign rank p-value = 0.01, ES = 2.3, statistical power = 0.9).

In the non-somatotopic case (TNS-1L), instead, no significant differences were found in the questionnaires. Even if the 1x6 Kruskal-Wallis on the PPS results reported a significant result for both the synchronous (H (5) = 15.03, p_SYNC_ = 0.01) and asynchronous (H (5) = 14.14, p_ASYNC_ = 0.015) case, the posthoc multiple comparisons placed the boundary of the PPS in the same location (between D5 and D6) for the asynchronous (from D5 all p_async_< 0.03) and the synchronous case (from D5 all p_sync_<0.02). All catch trials were never more than 2, indicating an appropriate accuracy of the subjects in detecting the tactile stimulus (S5 Fig in S1 File). Similarly, no significant dissimilarities are found in the MIT (t-test p = 0.64).

## Discussion

In this study, we proposed a new multisensory platform to induce the Full Body Illusion with a precise and controllable approach, combining Virtual Reality and Transcutaneous Electrical Nerve Stimulation. In this setup, we adopted a multifaceted measurement procedure integrating standard subjective and objective measurements. This allowed us to obtain more information on the strength of the illusion and on the validity of the adopted outcomes.

First of all, we reproduced the classical FBI approach to test the feasibility of the virtual environment combined with an experimenter-induced touch to generate the illusion. According to previous studies [11, 37, 38], we found that this method caused a higher subjective feeling of embodiment in the synchronous visuo-tactile stroking condition. However, the objective measurements were not in line with this finding as the PPS boundary and the MIT did not differ between the synchronous and asynchronous cases. It must be noted that previous studies have already raised concerns regarding the actual correlation between similar drift measurements and the illusion [18]. However, in this study, we used a recently proposed method [16] to compute the perceived location (i.e., Mental Imagery Task (MT)) that has been developed to overcome the limitations of the previous approach [11], criticized for its uncertain ability to measure the perceived mislocalization of the subject [16]. Nevertheless, even though the results did not reach a statistically significant difference between conditions, they showed a trend in favor of the synchronous one. Therefore, it is possible that with a larger sample size they would show the same MIT and PPS results previously reported [7, 19].

The urge of finding new methods to induce the FBI is not novel to the scientific field. Indeed, many researchers have developed alternative approaches to overcome the current issues. On one side, the need to find replicable and controlled ways to induce the illusion has led scientists to use VR combined with haptics, but these alternatives do not fully meet the requirements for an optimal solution: some do not adapt to the subject’s movements, others are heavy and not portable or require daunting set-ups with low-quality sensations elicited. Our platform replaced the typical somatosensory feedback used in FBI paradigms with an electrical nerve stimulation approach via TENS never been used before. The use of these technologies allows the platform to be very temporally precise. Indeed, the maximum latency of the visual (through VR) stimulation is 0.03 s [39] and of the electro-tactile one 0.016 s (calculated by measuring the difference in timestamps between the dispatch of the command to the stimulator and the reception of the acknowledge message). Therefore the max visuo-tactile latency could be 0.03 s. These delays would not be perceivable by the human senses, since an order of magnitude smaller than a response time of 0,1s which is perceived as instantaneous [40]. Moreover, they are well below the reaction time of humans to visual stimuli which is 0.26 s [41], which is the smallest imaginable for the in the case of the classical method to induce the classic FBI, therefore an order of magnitude less precise then the method here presented.

Our primary goal was to test whether the combination of these technologies could produce results coherent with literature. First, we used the platform with the visuo-tactile stimulation on the back trunk, one of the most used locations in the classical approach. We found that, as for the classical approach, the synchronous condition yielded significantly higher results in the subjective measurements. In addition, and differently from the classical FBI, the MIT resulted in a higher mislocalization of the subject in the synchronous case. However, the PPS did not follow the same trend, showing a larger boundary for the asynchronous condition. The reasons for this unusual finding may be related to the location of the stimulation and we refer to the third condition tested (TS-4L) to interpret this hypothesis. In the TS-4L condition, all the limbs of the subject were stimulated with TENS and we found that all the measurements yielded significant results in favor of the synchronous condition: the subjective outcomes indicated a higher embodiment, the PPS was expanded and the MIT was higher. It is well known that tactile sensitivity varies across our body, because of the different types and density of sensory afferents that innervate the skin; notably, hands and feet are more receptive to stimulation than back [42]. Therefore, this higher sensitivity may have influenced the illusion, supporting the use of TENS on limbs other than on the back to recreate the illusion.

The results of the TS-4L condition show that the illusion was successfully induced and confirmed by both subjective and objective measurements. When comparing this result to the classical approach (C-FBI) and with the results from other studies [7, 11, 19], it might be surprising that the C-FBI yielded no significant results in the objective measurements. However, we believe that—given the sample sizes of each group were equal—our multimodal platform seems to be able to induce a bigger effect size (ES_TS4L_ = 5.6 vs ES_C-FBI_ = 3.2) that may require fewer participants to see statistical differences.

Then, we explored whether stimulating only one limb could successfully induce the illusion. It is still a debate whether the sense of whole-body ownership arises even when stimulating only specific body parts. Previous studies have indirectly explored this issue by mechanically stimulating different body parts and measuring the ownership of the same stimulated and non-stimulated parts [28]. The finding that subjects perceived ownership also towards the non-stimulated areas suggested that the ownership “spreads” to incorporate the whole body; however, the explicit feeling of full body illusion was not measured. Very recent evidence comes from O’Kane and Ehrsson [30], who purposely designed a set-up where they stimulated one, two, or three limbs. Based on their results, they suggested that the feeling of ownership arises irrespective of the number of body parts being stimulated. However, this has never been explored with the use of TENS as haptic feedback.

Therefore, we replicated the illusion with our platform while stimulating only one body part (i.e., the foot). Once again, the results were significantly different between the conditions in favor of the synchronous one: higher embodiment by subjective reports expanded PPS and bigger MIT.

Finally, we wanted to test whether, in the case of only one limb being stimulated, the somatotopy (i.e., same stimuli location) was necessary. Spatial congruency has already been shown to be a crucial factor to elicit the illusion with previous approaches [12]. Likewise, with our platform, we found that visually stimulating the foot while delivering the somatosensory feedback to the hand does not elicit significant results: the subjective measurements yielded low and not significant differences, as well as the objective ones, which placed the PPS boundary in the same location and did not produce a mislocalization of subjects.

Although the present study provided several insights into a new approach to induce the FBI, several limitations may be noted. First, the duration of the experimental sessions was long (more than 2.5 hours) and might have impacted the results. However, to counterbalance possible fatigue effects we randomized the sequence of the conditions for each subject, as well as that of the outcome measures and subjects were allowed to take a break in between conditions. Moreover, we cannot completely exclude the possibility of demand characteristics’ influence on results, even if we standardize the experimental procedure and instructions given to participants and we randomized the conditions. Demand characteristics are cues, such as “prior beliefs, experimental instructions, and recruitment materials and aspects of the experimental procedure itself” [43], that influence the way the participant interprets the experiment.

It must be considered aldo that the outcome measures do not reflect all the heterogeneity of the approaches that could be used (e.g., heart rate, skin conductance). Future studies with a larger sample size could investigate other outcome measures, as well as the combination with movement tasks to provide further insights on the potential benefits that could be obtained. Finally, this study adopted a third-person perspective to induce the illusion, but there is evidence that a first person perspective might induce a stronger illusion [44].

Taken together, our results support the feasibility of our platform in inducing the FBI in a very reproducible manner and with a rigorous methodology that can overcome the limitations of other approaches. Furthermore, we highlight the potential future use of this system for rehabilitation purposes. First of all, such illusions have already been shown to be able to provide beneficial effects on pain perception [45], phantom sensations [46–48] and body image disturbance [48, 49]. Furthermore, manipulating the body schema has shown correlations with a variety of seemingly unrelated benefits, such as a reduction in the perceived weight of prosthetic devices and increased functional abilities in amputees [50–53]. Using our non-invasive, safe, and precise platform [54] with patients suffering from sensory loss who developed body disownership (e.g. stroke, amputation, somatoparaphrenia), will open up the possibility to elicit sensations in distal areas where patients lost their sensations, which would be impossible with existing platforms. Finally, peripheral nerve stimulation is a known therapeutic approach to treat neuropathic pain conditions [55, 56], hence the use of TENS as a substitution for other haptic feedback could yield beneficial effects also on pain relief.

In conclusion, our platform and our protocol stand as a valuable alternative to the current methods to induce the FBI, to obtain universal FBI guidelines that could boost the neuroscientific knowledge behind this phenomenon.

## Supporting information

S1 File Supporting information contains all the supporting tables and figures. (DOCX)

S1 Video Explanatory video of the experimental set-up and measures. The video explains the five different experimental conditions and the measurements that were taken during the experiment.
(MOV)
