# Supplementary material for: Reshaping the full body illusion through visuo-electro-tactile sensations
Source: PLoS One. 2023 Feb 1;18(2):e0280628. doi: 10.1371/journal.pone.0280628 (PMC9891501; doi:10.1371/journal.pone.0280628)
Supplement: S1 File — (DOCX) [file pone.0280628.s001.docx]

**Supplemental Inventory**

Supplemental Videos

Supplemental Materials and Methods

Supplemental Results

Supplemental References

**Supplemental Video**

Video S1: Explanation of the experimental conditions and measurements.

**Supplemental Materials and Methods**

# Experimental procedure

The total number of subjects (22) was divided in equally sized groups which took either 2 or 3 conditions, depending on the type and location of the stimulation.

Additionally, each condition was divided in 6 sessions: half of them consisted in 1 minute of stimulation to induce the illusion; the other half, instead, comprised the acquisition of 1 of the 3 outcome measures (with a different duration). A “stimulation” session was always alternated with a “metric” one, but the metrics order was randomized between conditions. The experimental procedure was designed as described to be able to acquire different measurements and avoid that during the last ones the illusion would fade away.

An example of one condition’s internal organization is shown below (Figure S1).


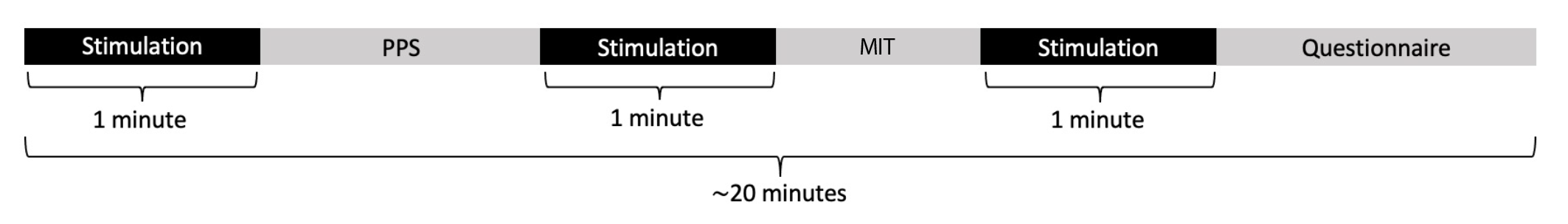


Figure S1: example of trials succession in a condition.

# Sensation calibration procedure

An example of the form that was filled during the calibration procedure is reported below (Figure S2).

During hands calibration the target nerve was primarily the median one, but when the elicited sensation did not spread enough electrodes were placed closer to the ulnar nerve.


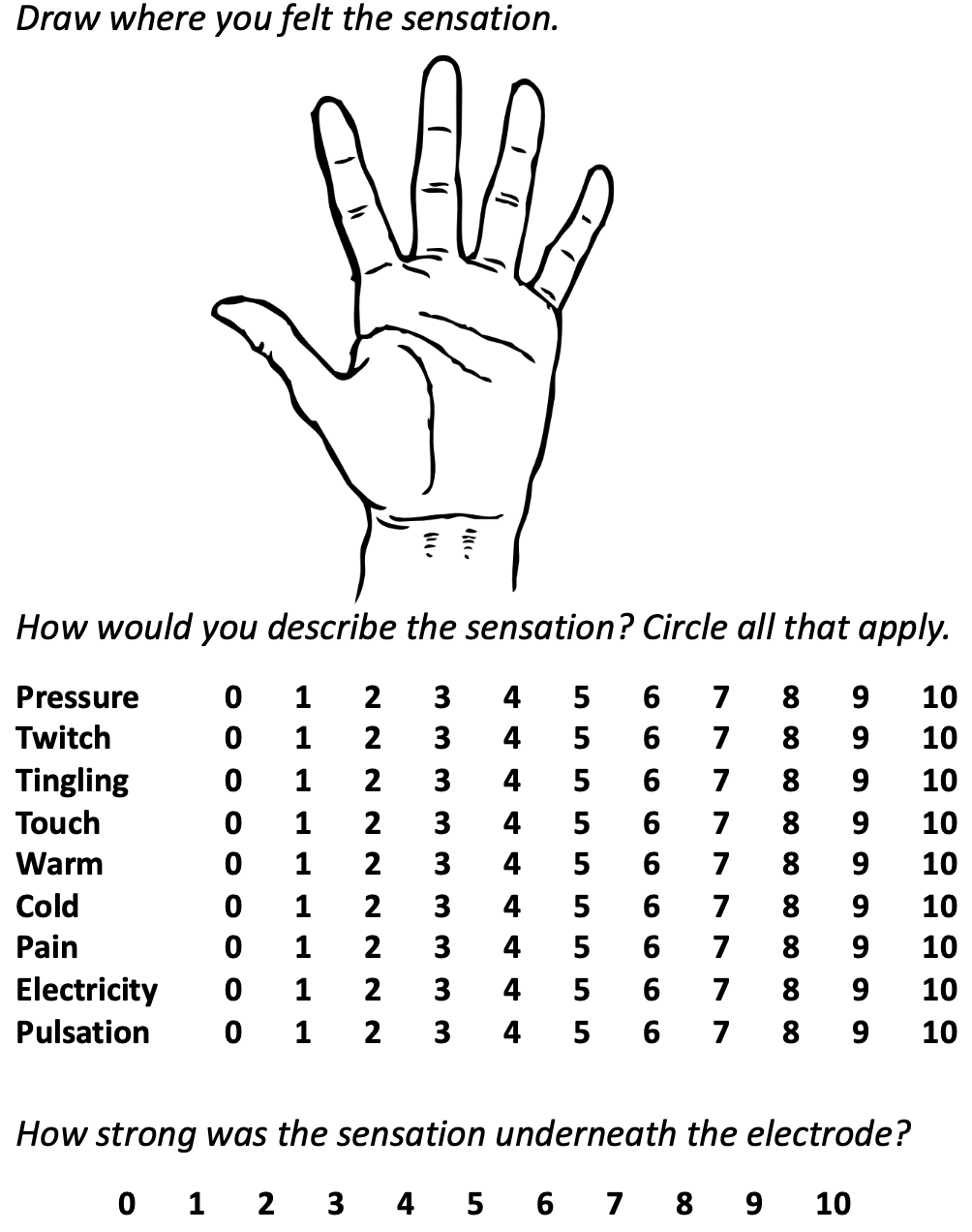


Figure S2: form filled from participants during the sensation calibration procedure.

# Outcome FBI measures

**Questionnaires**

All questionnaires’ items are reported in the following tables: Embodiment questionnaire (Table S1), Vividness and Prevalence questionnaire (Table S2) and PCI questionnaire (Table S3).

In the Embodiment questionnaire a further division is done between its main components: ownership, location, touch and controls (each one including 2 questions). Answers were given on a 7-points scale, from -3 (completely disagree) to +3 (completely agree), then re-scaled between 0 and 6.

In the Vividness and Prevalence questionnaire the 2 components are ranked differently: between 0 and 10 for vividness, between 0 and 100 for prevalence.

The PCI questionnaire is divided in 2 components: Body Image and Self-Awareness (each one made of 3 items). Each single element consisted of 2 opposite sentences with a 7-points scale (from 0 to 6) in between. The participant had to mark whether he agreed more with the left or right statement by choosing a grade closer to the former or to the latter (i.e., 0 means that he completely agreed with the left one).

Table S1: list of all questions belonging to the embodiment questionnaire filled at the end of each condition. Questions are grouped in 4 sub-categories (Ownership, Location, Touch and Control).

| **Embodiment questionnaire** |  |
| --- | --- |
| Component | Question |
| Ownership | Q1. I had the feeling that I was looking at my body |
|  | Q2. I felt as if the virtual body was my body |
| Location | Q3. I felt as if my body was located where I saw the virtual body |
|  | Q4. I felt as if my (real) body was drifting towards the virtual body |
| Touch | Q5. It seemed as if I felt the stimulation in the location where I saw the virtual body stimulated |
|  | Q6. It seemed as if the tactile sensation I felt was caused by the visual stimulus on the virtual body |
| Control | Q7. It seemed as if I might have more than one body |
|  | Q8. It seemed as if the stimulation I felt was located somewhere between my physical body and the virtual body |

Table S2: list of the vividness and prevalence questions.

| **Vividness and Prevalence questionnaire** |  |
| --- | --- |
| Component | Question |
| Vividness | Q9. Using a scale from 0 to 10 quantify the vividness: how much the illusion of being outside your body was realistic? |
| Prevalence | Q10. Using a scale from 0 to 100% quantify how long you had the illusion of being outside your body |

Table S3: list of all itmes belonging to the PCI questionnaire. Questions are grouped in 2 sub-categories (Body-Image and Self-Awareness).

| **PCI questionnaire** |  |  |
| --- | --- | --- |
| Component | Left statement | Right statement |
| Body Image | My body ended at the boundary between my skin and the world | I felt my body greatly expanded beyond the boundaries of my skin |
|  | My bodily feelings seemed to expand into the world around me | My bodily feelings were confined to the area within my skin |
|  | I continually maintained a very strong sense of separation between myself and the environment | I experienced intense unity with the world; the boundaries between me and the environment dissolved away |
| Self-Awareness | I was not aware of being aware of myself at all; I had no self-awareness | I was very aware of being aware of myself; my self-awareness was intense |
|  | I was continually conscious and well aware of myself | I lost consciousness of myself |
|  | I maintained a very strong sense of self-awareness the whole time | I did not maintain a very strong sense of self-awareness at all |

**Peri-Personal Space (PPS)**

Method

During the acquisition of this metric subjects received an electrical pulse of *100 ms* while looking at a tennis ball looming towards them. The task was to press the button on the controller they were holding as soon as they felt the pulse.

The total distance between the starting point of the ball and the subject was *2.40 meters*, divided in 6 equally-sized slots of *0.4 meters*, labelled from D1 to D6, with D1 being the one closer to the subject (Figure S3).


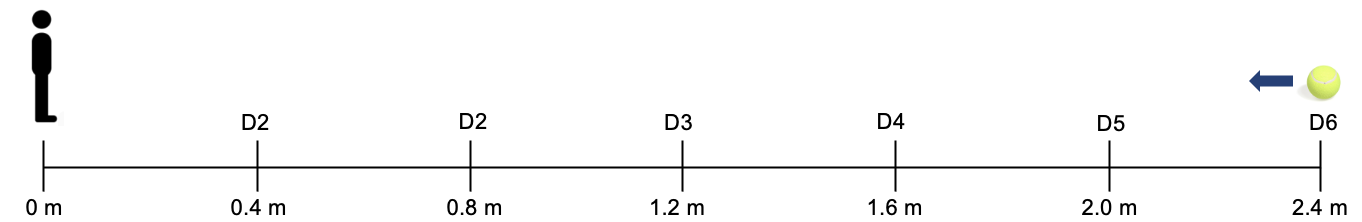


Figure S3: scheme of the PPS metric acquisition. Distances from D1 to D6 identify the possible locations of the ball when the electrical pulse is delivered to the participant.

The electrical pulse was delivered when the ball was at one of the 6 distances. For each distance 10 acquisitions were carried out for a total of 60 reaction times (RTs) registered for each condition.

In addition, baseline trials (RT to the sole electrical pulse, without the ball) were collected for distances D1 and D6; 10 acquisitions for both distances were recorded as well, for a total of 20 RTs. Then, a single baseline was computed for every subject in each condition: it was done evaluating the mean RT of both D1 and D6 and taking the smallest one.

Lastly, 10 catch trials were added: in these cases, the ball was present, but no electrical stimulation was delivered to the subject who, in fact, was not supposed to press the button. Their aim was to test his level of attention.

A total of 90 RTs was collected, randomizing the order of the different types of trials.

Two different kinds of PPS were acquired depending on the condition: peri-foot or peri-trunk PPS[1]. The former was measured in case the illusion was induced with a stimulation localized to the foot (TS-1L and TNS-1L): the electrical pulse was delivered to the stimulated foot and the ball was looming towards it. The latter was measured in case the stimulation was localized either on the back or on the 4 limbs (c-FBI, T-FBI and TS-4L): the electrical pulse was delivered to the right hand and the ball was looming towards the participant’s face.

The fact that different body-part specific PPS representations exist has been already discussed in literature[1], stating that a difference in extension exist between them even if they are not fully independent from each other.

Statistical analysis

For each different trial and distance, the 10 repeated measures were averaged. So, for a single condition, 6 experimental RTs (one per distance) and 1 baseline RT (considering the fastest between the two, as explained above) were available per subject.

The statistical analysis of a single condition consisted of 6 t-test comparisons between the baseline RT and the 6 experimental RTs: each distance was considered inside the PPS region if the experimental RT was significantly faster than baseline, outside otherwise.

The analysis of catch trials was purely qualitative, considering that a reaction in those cases was index of distraction from the task.

**Mental Imagery Task**

Method

For the computation of this metric, the avatar was removed from the room and a ball was placed on the floor *3 meters* ahead the subject. The ball rolled towards him with a constant velocity of *0.4 m/s*. After *3 s* the HDMI screen became black and he was required to press the button on the controller when he imagined the ball was crossing his feet. The ball’s position at the instant the button was pressed by the subject represented his perceived self-location (i.e., the position where he located his feet).

For each condition a total of 6 acquisitions were carried out.

Additionally, 6 pre-test measures were performed before starting the experiment, when the subject was completely unaware of the avatar. These were then taken as self-localization baseline.

Statistical analysis

For each participant, one MIT measure was obtained per condition averaging the 6 collected repetitions. The baseline value, instead, was one, computed as mean of the 6 pre-test values. Experimental data were then normalized by subtracting the baseline.

Statistical comparisons consisted of a t-test between the synchronous and asynchronous case to check whether a significant difference was present.

**Supplementary Results**

**Questionnaires**

As previously said, embodiment questionnaire was divided in 3 sub-sections: ownership, location and touch. In Figure S4 detailed results are reported.


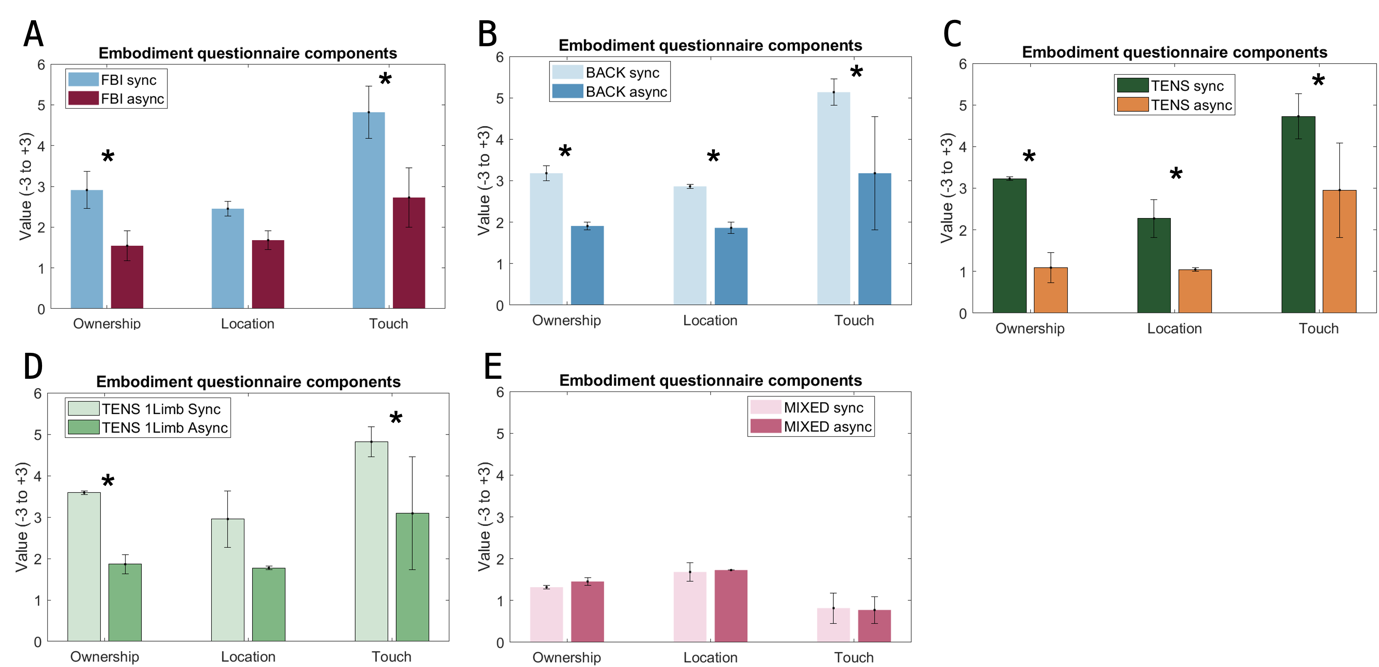


Figure S4: results of the embodiment questionnaire divided in the 3 components (Ownership, Location and Touch). Each panel is relative to a different condition: C-FBI (Panel A), T-FBI (Panel B), TS-4L (Panel C), TS-1L (Panel D), TNS-1L (Panel E).

In the classical FBI condition (C-FBI) (Figure S4 A) the synchronous condition was rated as significantly higher compared to the asynchronous for Ownership and Touch components; for Location the value was higher for the synchronous case but significance was not reached (Own_SYNC_ = 2.91 ± 0.45, Own_ASYNC_ = 1.55 ± 0.36, Wilkoxon signrank p=0.023; Loc_SYNC_ = 2.45 ± 0.18, Loc_ASYNC_ = 1.68 ± 0.23, Wilkoxon signrank p=0.093; Touch_SYNC_ = 4.82 ± 0.64, Touch_ASYNC_ = 2.73 ± 0.73, Wilkoxon signrank p=0.004).

In the back stimulation condition (T-FBI) (Figure S4 B) the synchronous condition was rated as significantly higher compared to the asynchronous for all the 3 embodiment components (Own_SYNC_ = 3.18 ± 0.18, Own_ASYNC_ = 1.91 ± 0.09, Wilkoxon signrank p=0.009; Loc_SYNC_ = 2.86 ± 0.05, Loc_ASYNC_ = 1.86 ± 0.14, Wilkoxon signrank p=0.002; Touch_SYNC_ = 5.14 ± 0.32, Touch_ASYNC_ = 3.18 ± 1.36, Wilkoxon signrank p=0.004).

In the somatotopic 4Limbs TENS condition (TS-4L) (Figure S4 C) the synchronous condition was rated as significantly higher compared to the asynchronous for all the 3 embodiment components (Own_SYNC_ = 3.22 ± 0.05, Own_ASYNC_ = 1.09 ± 0.36, Wilkoxon signrank p=0.002; Loc_SYNC_ = 2.27 ± 0.45, Loc_ASYNC_ = 1.05 ± 0.04, Wilkoxon signrank p=0.004; Touch_SYNC_ = 4.73 ± 0.55, Touch_ASYNC_ = 2.95 ± 1.14, Wilkoxon signrank p=0.023).

In the somatotopic 1Limb TENS condition (TS-1L) (Figure S4 D) the synchronous condition was rated as significantly higher compared to the asynchronous for Ownership and Touch components; for Location the value was higher for the synchronous case but significance was not reached (Own_SYNC_ = 3.59 ± 0.05, Own_ASYNC_ = 1.86 ± 0.22, Wilkoxon signrank p=0.002; Loc_SYNC_ = 2.95 ± 0.68, Loc_ASYNC_ = 1.77 ± 0.04, Wilkoxon signrank p=0.080; Touch_SYNC_ = 4.82 ± 0.36, Touch_ASYNC_ = 3.09 ± 1.36, Wilkoxon signrank p=0.035).

In the non-somatotopic 1Limb TENS condition (TNS-1L) (Figure S4 E) the synchronous and asynchronous conditions were not significantly different for all the 3 embodiment components (Own_SYNC_ = 1.31 ± 0.04, Own_ASYNC_ = 1.45 ± 0.09, Wilkoxon signrank p=0.797; Loc_SYNC_ = 1.68 ± 0.23, Loc_ASYNC_ = 1.73 ± 0.0, Wilkoxon signrank p=0.937; Touch_SYNC_ = 0.82 ± 0.36, Touch_ASYNC_ = 0.77 ± 1.31, Wilkoxon signrank p=0.992).

**Peri-Personal Space (PPS)**

PPS results reported in the main text only consider experimental and baseline trials. Those relative to catch trials were not used for statistical analysis but only for a qualitative one, as index of subjective attention. In fact, as no electrical pulses were delivered in these trials, participants were not supposed to react.

Figure S5 shows the bar plots for the 2 participants’ groups are reported, representing the number of times (over 10 repetitions) participants reacted.

**
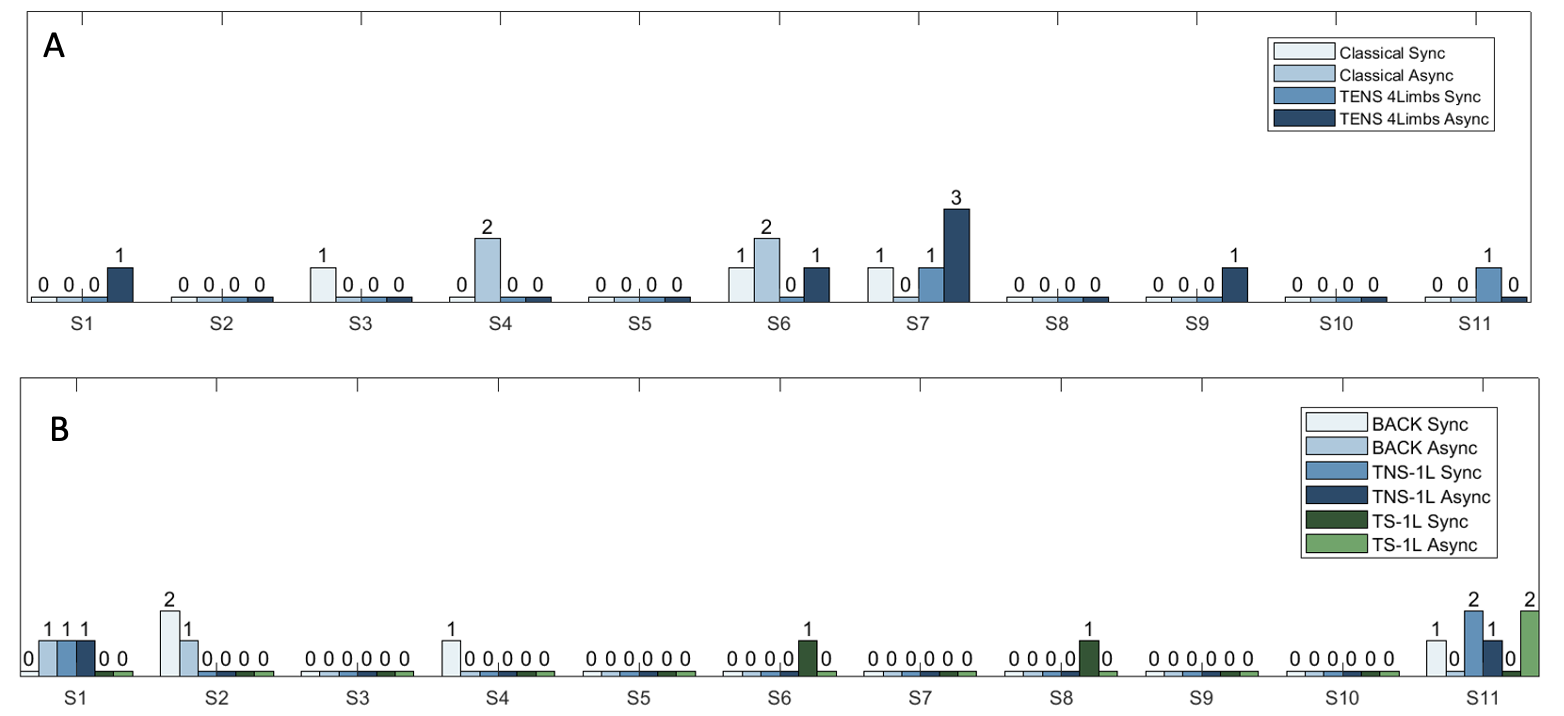
**

Figure S5: number of answers given by participants during PPS catch trials. Panel A is relative to the first group of subjects (who took C-FBI and TS-4L conditions); Panel B is relative to the second group (who took T-FBI, TS-1L and TNS-1L conditions). On the x-axis there is the subject number and results are reported divided per condition.

**Supplementary References**

1. Serino A, Noel J-P, Galli G, Canzoneri E, Marmaroli P, Lissek H, et al. Body part-centered and full body-centered peripersonal space representations. Sci Rep. 2015;5: 18603. doi:10.1038/srep18603
